# Evaluation of the reliability of markerless tumor tracking with single‐energy and dual‐energy imaging using machine learning

**DOI:** 10.1002/acm2.70621

**Published:** 2026-05-21

**Authors:** Ha Nguyen, Jason Luce, Liangjia Zhu, Mathias Lehmann, Hyejoo Kang, Matthew M. Harkenrider, John C. Roeske

**Affiliations:** ^1^ Department of Radiation Oncology Stritch School of Medicine Cardinal Bernadin Cancer Center Loyola University Chicago Maywood Illinois USA; ^2^ Varian Medical System Palo Alto California USA; ^3^ Department of Radiation Oncology Loyola University Medical School Maywood Illinois USA; ^4^ Department of Radiation Oncology Mayo Clinic Phoenix Arizona USA

**Keywords:** image‐guided radiation therapy, machine learning, radiation, tumor motion tracking

## Abstract

**Background:**

Markerless tumor tracking (MTT) using single‐energy (SE) kilovoltage (kV) imaging has been proposed as a technique for lung tumor motion management. However, bony structures can obscure the tumor and make tracking challenging. Dual‐energy (DE) subtraction imaging can suppress the bone and therefore enhance tumor visibility, potentially improving tracking accuracy.

**Purpose:**

To develop a machine learning model that predicts the reliability of MTT for SE and DE imaging using phantom and patient data.

**Methods:**

Images were collected at high and low energy (120/60 kV) using fast‐kV switching on dynamic thorax phantom with 5–15 diameter targets and 20 lung cancer patients, including one patient with two tumor locations. DE images were then generated off‐line using weighted logarithmic subtraction. Separately, a template‐based tracking algorithm was used to track the tumor on both phantom and patient images. For phantom data, the ground truth (GT) was defined by the cos^4^ waveform programmed into the motion controller. Since the GT was unknown for patient data, it was estimated using a Kalman filter and further refined using visual inspection. The tracking success rate (TSR) was subsequently calculated. Based on TSR, patients were stratified into three tracking groups: good, moderate, and poor. A logistic regression (LR) model was implemented with tracking features, including match score, peak to side‐peak ratio (PSR), *x*‐ and *y*‐velocity. To address the distribution differences between phantom and patient data, a domain indicator and interaction features were also incorporated. The model was trained on phantom and 17 patient data using leave‐one‐out cross‐validation, and its performance was evaluated on three held‐out patients, one from each tracking group. Separate LR models were developed for each imaging modality, resulting in SE‐LR and DE‐LR models. For each held‐out patient, the confusion matrix, sensitivity, and specificity were reported.

**Results:**

For both SE‐LR and DE‐LR models, PSR has the largest coefficient value, indicating that it is the most influential factor when determining the reliability of tracking. At a specificity of 95%, the DE‐LR model achieved significantly higher sensitivity than the SE‐LR model (0.908 vs. 0.731, *p* < 0.01 for phantom data and 0.657 vs. 0.589, *p* < 0.01 for patient data). In the three held‐out patients, the DE‐LR model outperformed the SE‐LR model across all metrics. The largest gains were found in the patient with good tracking quality.

**Conclusions:**

The proposed LR models successfully predicted the reliability of SE‐ and DE‐MTT. Across phantom and patient datasets, the DE‐LR model consistently demonstrated improved performance compared with the SE‐LR model, suggesting that DE imaging may be beneficial for MTT applications.

## 1 INTRODUCTION

Markerless tumor tracking (MTT) is a technique being considered in radiation therapy (RT) to monitor the position of a tumor without the need for implanted fiducial markers.^1^, ^2^ This approach aims to improve the accuracy of radiation delivery, especially for tumors affected by respiratory motion, such as those in the lungs. On a linear accelerator with an on‐board imager (OBI), MTT often relies on single‐energy (SE) x‐ray imaging to visualize the tumor.^1^ However, SE‐MTT can be challenging due to overlying bony structures, which can lead to inferior tumor visibility.^2^, ^3^

Dual‐energy (DE)‐MTT offers a solution to some of the limitations of SE‐MTT. By acquiring images at two different x‐ray energies and performing a weighted logarithmic subtraction (WLS) of these image pairs, DE imaging can suppress bone and enhance the visibility of soft tissues, including the tumor.^1^, ^3^ This improved tumor visibility can potentially increase the accuracy of MTT. Several studies have indicated that DE imaging can enhance lung tumor visualization and provide accurate intrafractional imaging for MTT. In phantom studies, DE imaging reduced the bone signal while enhancing tumor detectability, leading to a higher tracking accuracy compared to SE imaging.^3^ In early clinical evaluations of SBRT patients, DE imaging substantially improved overall tracking performance, increasing the fraction of successfully tracked frames from 60.3% with SE to 93.4% with DE.^4^ In a separate clinical study, DE imaging reduced the percentage of frames that could not be tracked, relative to SE, from 4.0% to 1.4% while reducing the mean tracking error from 2.21 ± 2.38 to 1.78 ± 2.02 mm.^5^ Altogether, phantom and patient data consistently demonstrated that DE imaging enhances tumor visualization and tracking accuracy over SE imaging alone.

Despite these advancements, a major challenge in validating MTT systems, particularly in patient data, is the lack of ground truth (GT). The true tumor position during treatment is rarely known with precision, complicating the evaluation of tracking accuracy. Anthropomorphic phantom studies can be programmed to produce GT trajectories. However, phantoms do not capture patient‐specific deformations and tissue variability, limiting their generalizability.^6^, ^7^ Even in promising clinical studies using kV fluoroscopy, reported tumor trajectories must often be inferred or correlated with external surrogates rather than validated directly against known positions.^8^, ^9^, ^10^ This uncertainty raises the essential question: How trustworthy is any given tracked data point during treatment? Given the lack of GT in patients, assessing the reliability of MTT becomes essential. A reliability measure provides a surrogate indicator of tracking confidence, ensuring that only reliable tracking is used for clinical decision making and treatment delivery. This is critical because the reliability of tumor tracking has direct clinical consequences. If tracking is incorrect, the delivered dose might miss the tumor or irradiate surrounding healthy tissue, undermining both efficacy and safety of radiotherapy.

Machine learning (ML) models have demonstrated considerable success in many medical studies,^11^, ^12^, ^13^, ^14^ including MTT research.^15^, ^16^, ^17^ For instance, a Siamese neural network was trained using digitally reconstructed radiographs (DRRs) from planning CT scans to detect tumor locations.^16^ The proposed model produced a 100% tracking rate, compared to 62%–82% by conventional template matching techniques.^16^ In another study, Yu and colleagues proposed the use of a conditional generative adversarial network (Pix2Pix) to generate synthetic decomposed target images (sDTI) from real‐time kV images, which increased tumor contrast and enabled more accurate template‐matching‐based tracking.^15^ Of note, these prior MTT studies have focused primarily on improving tumor localization performance, whereas the problem of determining whether the predicted tumor position can be trusted has not been addressed. To fill this gap, we developed and evaluated an ML model that can accurately predict the reliability of both SE‐ and DE‐MTT, providing a confidence metric for MTT during RT. The proposed approach may enable clinicians to identify trustworthy tracked tumor positions and make more informed treatment decisions.

## 2 METHODOLOGY

**FIGURE 1:**
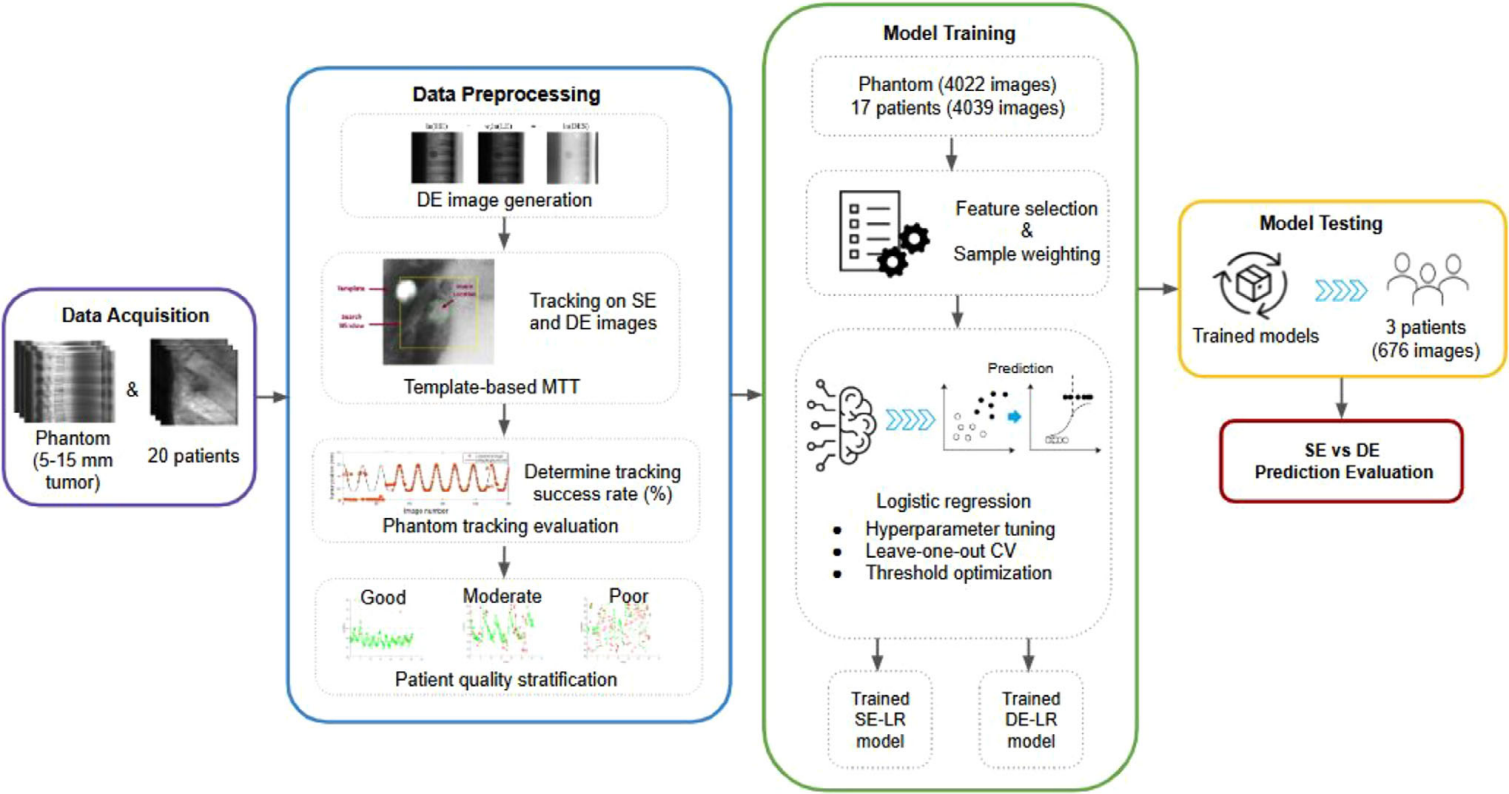
Workflow of the machine learning process for evaluating the reliability of SE and DE‐MTT. CV, cross‐validation; DE, dual energy; LR, logistic regression; MTT, markerless tumor tracking; SE, single energy.

Figure 1 presents an overall workflow of the study. First, images were collected of a dynamic thorax phantom with 5–15 mm simulated tumors and separately from 20 patients with early‐stage lung tumors, including one patient with two tumor sites. Data processing was then performed, including generation of DE images, template‐based tracking on both SE and DE images, and evaluation of phantom and patient tracking quality. The next step involved developing a logistic regression (LR) model to predict the accuracy of SE‐ and DE‐MTT. This model was trained on both phantom and patient data. Lastly, the trained SE‐LR and DE‐LR models were tested on held‐out patients, and the model performance of each modality was evaluated. A more detailed description of each step is outlined below.

### 2.1 Data acquisition

Images were collected using the fast‐kV switching functionality of the OBI on a TrueBeam linear accelerator (Varian Medical Systems, Palo Alto, CA). The fast‐kV switching firmware delivers consecutive x‐ray pulses that alternate between programmed energies, accessed via Developer Mode in TrueBeam software version 2.7MR3 and higher.^18^ In this study, alternating 60 kV (60 mA, 20 ms) and 120 kV (15 mA, 20 ms) were used with a frame rate of 15 frames per second (fps). This results in DE images with an effective frame rate of 7.5 fps.

Phantom data were acquired using the CIRS dynamic thorax motion phantom (CIRS Inc., Norfolk, VA), which mimics an average human thorax and includes anatomical features such as two lung cavities, ribs, and spine. One of the lung‐equivalent cavities contains a cylindrical insert powered by a motor and actuator. Inside this insert, spherical simulated tumors, ranging from 5 ‐15 mm in 5 mm increments, can be placed individually. Respiratory motion was simulated by actuating the cylindrical insert along the superior–inferior direction using a programmable motion sequence (cos^4^(*x*) waveform).^1^, ^2^ Three clinically relevant motion scenarios were evaluated: stationary (simulating breath‐hold), slow breathing (amplitude = 7.5 mm, period = 5 s), and fast breathing (amplitude = 7.5 mm, period = 2.5 s).^2^

Separately, 20 early‐stage lung cancer patients undergoing SBRT were included in this study. A summary of patient and tumor characteristics is provided in Table 1. A more detailed description of patient and tumor characteristics is described elsewhere.^19^ Briefly, all patients were positioned in a supine orientation and immobilized using an alpha cradle indexed to the treatment table. 4D‐CT scans were acquired for each patient using our departmental CT scanner (Open AS, Siemens Healthineers) and exported to the treatment planning software (Eclipse, Varian Medical Systems, Palo Alto, CA) for the contouring of the internal target volume (ITV). Treatment planning was performed following our standard protocols. In general, an overall prescription dose of 50–60 Gy was delivered in 3–5 fractions to the planning target volume (PTV).^20^ At the completion of one of the treatment fractions, a sequence of images over a 180° arc was acquired using a fast‐kV switching functionality of the OBI via Developer Mode. The imaging parameters for the patient cohort were consistent with those employed in the phantom study. All images were obtained with the tumor positioned at the isocenter, as defined by the treatment plan, and with a source‐to‐detector distance (SDD) of 150 cm.

**TABLE 1:** Summary of patient and tumor characteristics.

Characteristics	Number (*N*)
Sex	
Male	11
Female	9

Laterality	
Right	12
Left	9

Gross tumor volume	
Volume range	0.8–24.3 cc
*N* < 5 cc	12
*N* ≥ 5 cc	9

### 2.2 Dual‐energy image generation

**FIGURE 2:**
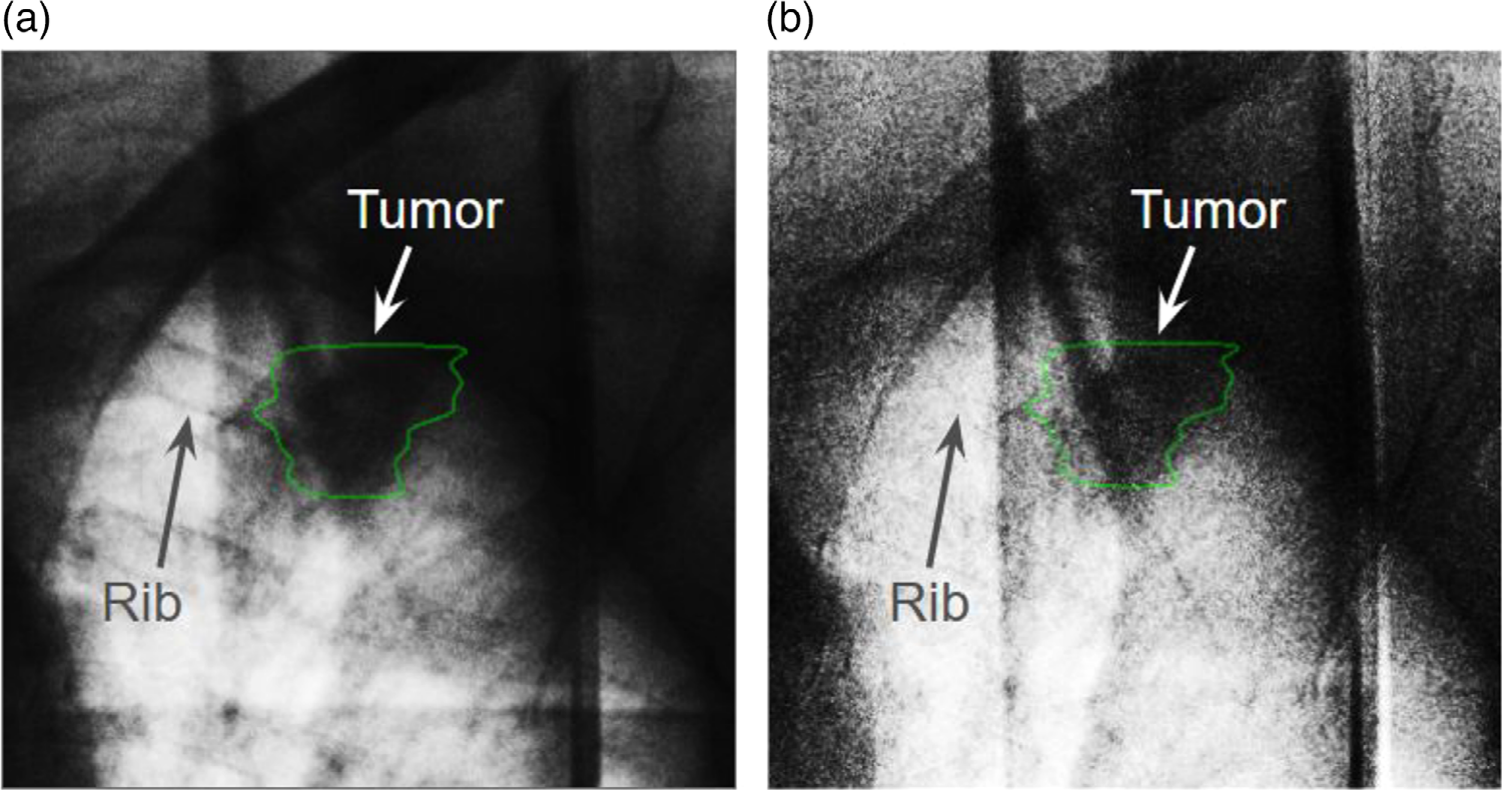
(a) Single energy (SE) and (b) dual energy (DE) subtraction images of Patient 5. When the tumor and rib overlapped, DE imaging suppressed the bone and enhanced tumor visibility. The green contour represents the tumor.

The creation of bone‐suppressed DE images using WLS was performed off‐line using in‐house software in MATLAB R2023b (MathWorks, Natrick, MA, USA). Each pair of 60/120 kV images was used to generate the DE images through the following equation^1^, ^18^

(1) $$\mathrm{DE}=\ln\left(I_{H}\right)\;-\;w_{s}\ln\left(I_{L}\right)$$

where *I*_H_ and I_L_ are the intensities of individual pixels on the high‐energy (HE) and low‐energy (LE) projections, respectively, and *w_s_* is the weighting factor to produce the bone‐suppressed DE image.^1^, ^18^ The values of *w_s_* were 0.69 and 0.42 for phantom and patient images, respectively, based on previous studies.^1^, ^21^ While fast‐kV switching reduces subsequent motion artifacts in DE images, it does not completely eliminate them. A small delay of ∼67 ms (∼0.4° gantry rotation) exists between HE and LE acquisitions, which can introduce misregistration artifacts in the resultant DE images. Prior to DE image generation, a rigid image registration based on mutual information was applied to align the HE and LE images to eliminate these artifacts.^2^, ^18^ From this point onward, the HE image is referred to as SE. This simplification is used because, in the context of DE imaging, the HE acquisition corresponds to SE x‐ray image used clinically. An example of SE and DE images from a representative patient is shown in Figure 2.

### 2.3 Markerless tumor tracking

MTT was performed offline using a template‐based algorithm in a non‐commercial research software (RapidTrack v3.0, Varian medical System, Palo Alto, CA). A 2D tumor template is first derived from the contoured tumor on the planning CT scan. As the template is iterated over the search window, the tracking algorithm computes the normalized cross‐correlation (NCC) between each image and the template, producing a match score surface.^22^ The peak of the match score surface is identified as the predicted tumor location. The confidence of this peak is quantified as the peak‐to‐side lobe ratio (PSR) from the match score surface.^22^

For phantom data, the GT tumor trajectory was defined by the cos^4^ (*x*) waveform programmed into the motion controller. The tumor tracking success rate (TSR) is defined as the percentage of images in which the differences between the predicted and GT tumor location were < 2 mm. Note that the threshold for MTT is not well defined; however, 2 mm has been widely used in previous MTT studies.^6^, ^20^, ^21^, ^22^ For patient data, the GT of the tumor location is unknown. Therefore, Bayesian inference based on a Kalman filter (KF) with constant acceleration was employed to determine the estimated GT (eGT).^8^, ^20^, ^23^, ^24^ The KF is a robust and effective estimator for a dynamical system's position even when measurement errors are present. It functions as a statistical inference method that refines the estimated true position of an object by integrating predicted and measured positions. This approach has been widely utilized in previous research to estimate the 3D position of tumors in clinical data.^23^ In this study, the KF was utilized to estimate GT through an iterative two‐step process of prediction and correction. In brief, the prediction step forecasts the tumor position before a new measurement is taken. This is achieved using a state transition model that projects the tumor's motion from the previous frame to the current one. The correction step then refines the predicted position using the current measurement. The updated, and more accurate, estimated position is considered as the GT. Details about the estimation of GT using KF for MTT can be found elsewhere.^20^

Although KF is a powerful tool for estimating a dynamic system's state, it has inherent limitations when the tracking exhibits a non‐linear or abrupt motion.^25^, ^26^ When a tumor undergoes a sudden and significant change in direction or speed, the filter is unable to adapt to this abrupt motion. Thus, visual inspection was incorporated to further refine the patient eGT. This step leverages a human observer's ability to recognize non‐linear patterns of tumor motion that are not captured by the KF's predictive model. In our case, the eGT generated by KF was visually inspected by two reviewers. Similar to the phantom data, TSR was calculated for patient tracking results.

To evaluate patient tracking quality, we established baseline values using the average TSRs from the phantom data. The average TSRs were 47.6%, 72.2%, and 84.2% for 5, 10, and 15 mm tumors, respectively. Based on the midpoints between these values, a three‐level tracking quality system was defined. The midpoint between the 5 and 10 mm TSRs (59.9%) and the midpoint between the 10 and 15 mm TSRs (78.2%) were selected as thresholds. Accordingly, patient tracking quality was stratified as poor (TSR < 59.5%), moderate (59.5% ≤ TSR ≤ 78.0%), and good (TSR > 78.0%) based on these criteria.

### 2.4 Machine learning model

In this study, an LR model was implemented to predict the reliability of tracking results. LR is a classification technique that uses a sigmoid function on the weighted linear combination of input features, generating output value between 0 and 1 that represents the probability of the event occurring.^27^, ^28^ A detailed description of the proposed model is provided below.

#### 2.4.1 Tracking features, domain indicator, and interaction terms

The model was developed using features derived from the tracking data, including match score, PSR, and *x* and *y*‐direction velocities. Match score and PSR have been previously used as predictors of successful template matching.^4^, ^7^, ^29^ Higher values of both metrics generally indicate more accurate and reliable tracking. In addition to these similarity metrics, the *x* and *y*‐positions of the tracked tumor were used to compute velocity in each direction. Velocity features were incorporated to capture the temporal smoothness of motion, as abrupt frame‐to‐frame displacements often signify unreliable tracking. Velocity was chosen over higher‐order derivatives because numerical differentiation of position data tends to amplify noise and yield unstable estimates, thereby reducing feature reliability.^30^, ^31^ Collectively, these features were selected as the input features of the model. Match score was standardized by subtracting the mean and dividing the standard deviation (standard scaler). PSR, *x* and *y* velocities, which were more prone to outliers,^32^ were scaled using the median and interquartile range (robust scaler), making the transformation less sensitive to extreme values.

Generally, it is important to note that phantom and patient data differ due to multiple factors, such as motion and anatomical variation,^33^, ^34^ which lead to distributional differences in image characteristic.^35^ These differences cause variations in match score and PSR, resulting in statistical distribution mismatches between phantom and patient datasets. This kind of discrepancy between domains is well‐described in the ML literature as one of the major challenges in medical research.^35^, ^36^ Domain adaptation techniques are often used to address the distribution differences between datasets.^37^ In this study, the domain indicator (DI) was included as a feature, coded as phantom = 0 and patient = 1, thereby establishing the phantom data as the baseline against which the patient data is compared. That is, the phantom, representing a controlled and static environment, acts as the source domain, while the patient, with inherent motion and physiological variability, represents the target domain.^38^

The interaction features were then incorporated through a DI variable to differentiate between phantom and patient data.^39^ By multiplying each tracking feature with this indicator, the model learns distinct coefficients for patient data, effectively adapting these from the phantom baseline. This strategy has been widely used to handle domain adaptation between source and target datasets in biomedical modeling and transfer learning.^37^, ^39^ Overall, DI and interaction features enabled the model to adjust to the phantom and patient domain accordingly.

In this study, the LR model^40^, ^41^ can be expressed as follows:

(2) $$P\left(x\right)\;=\;\frac{1}{1+e^{-X}}$$

where *P*(*x*) is the probability of tracking reliability, and *X* is the combination of independent features. These features are expressed as follows: $X=\beta_{0}+\beta_{1}x_{1}+\beta_{2}x_{2}+\beta_{3}x_{3}+\beta_{4}x_{4}+\beta_{5}\text{DI}+\beta_{6}\left(\text{DI}\times x_{1}\right)+\beta_{7}\left(\text{DI}\times x_{2}\right)+\beta_{8}\left(\text{DI}\times x_{3}\right)+\beta_{9}\left(\text{DI}\times x_{4}\right)$ where *β*_0_ is the intercept; *β*_1_–*β*_9_ are coefficients of features; *x*_1_–*x*_4_ are tracking variables, including match score, PSR, *x*‐ and *y*‐velocities, respectively; and DI is a domain indicator. The first five terms describe the phantom baseline model (DI = 0). The next five terms, including the DI and interaction features, are available when the model is applied to patient data (DI = 1).

#### 2.4.2 Sample weighting and hyperparameter tuning

The study incorporated a sample weighting scheme to influence the learning process based on data quality.^42^, ^43^, ^44^ The sample weighting for patients with moderate and poor tracking was optimized using iterative methods. The weighting factors were swept across ranges of 0.4–0.8 for moderate tracking patients and 0.1–0.3 for poor tracking patients. The final weighting factors selected were 0.6 for moderate and 0.2 for poor tracking, with good tracking patient data having a weight of 1. This scheme was designed to allow the model to learn primarily from high‐confidence samples, which are patients with good tracking data.^43^, ^44^ The data from moderate and poor tracking patients was still considered, but with a lesser impact on the model's training due to its lower assigned weight.^43^ This approach ensured that the model's learning was prioritized toward tracking data with high confidence, resulting in a more robust and trustworthy assessment of tracking reliability.

In this study, the total loss function of the LR model includes a cross‐entropy function and regularization penalty terms^45^, as follows:

(3) $$L_{\mathrm{Total}}=-\frac{1}{N}\sum_{i-1}^{N}\left[y_{i}\log{\hat{y}}_{i}+\left(1-y_{i}\right)\log\left(1-{\hat{y}}_{i}\right)\right]+\lambda R\left(w\right)$$

where *N* is the number of samples, $y_{i}\in \left\{0,1\right\}$ is the GT, ${\hat{y}}_{i}=P\left(x\right)$ is the predicted probability from LR, λ is the regularization strength, w is the model coefficient, and R(w) is the regularization penalty term.^45^ To prevent overfitting, regularization terms are added to penalize large coefficients, which helps the model generalize better to unseen data.^45^, ^46^ The strength of this penalty is controlled by λ. A large value of λ corresponds to strong regularization, forcing the model to be simpler and reducing the risk of overfitting. There are three types of regularization penalties: L1, L2, and elastic net.^45^ L1 regularization ($R\left(w\right)=\sum |w_{j}|$) adds a penalty proportion to the absolute value of the coefficients, whereas L2 regularization ($R\left(w\right)=\sum w_{j}^{2}$) adds a penalty proportion to the squared magnitude of the coefficients. Elastic net is a hybrid approach that combines both L1 and L2 penalties.^47^ L1_ratio hyperparameter controls the linear combination of the L1 and L2 penalties. Two different solvers, liblinear and saga, were also optimized to compute the optimal coefficients for the LR model by minimizing the loss function. Liblinear is a well‐established and fast solver, particularly effective for smaller datasets and for models using L1 and L2 penalties, whereas saga is a stochastic average gradient descent method that can handle all three types of penalties.^46^ These hyperparameters, including λ, penalties, L1_ratio, and solvers, were tuned iteratively to maximize the model's performance.

#### 2.4.3 Model training

The study trained a separate LR model for each imaging modality—SE and DE. Specifically, tracking data from the phantom and 17 patients were used for model training, and 3 patients were held out for final testing. Due to the small sample size, a leave‐one‐out (LOO) cross validation strategy was employed.^48^, ^49^ This method is a form of cross‐validation where all tracking data from a single dataset is held out as the validation set in each fold. The model is trained on the remaining data and then evaluated on the validation set. This process is repeated until every dataset has served as the validation set exactly once. The LOO strategy prevents data leakage and ensures an unbiased assessment of the model's performance.^48^ The cut‐off threshold was also optimized using the training data to achieve a specificity of 95%. The decision to target a high specificity is to minimize false positives, as incorrectly classifying unreliable tracking as reliable could lead to critical errors in the clinical setting.^2^ In our case, sensitivity reflects the model's ability to correctly recognize reliable tracking results as reliable, while specificity refers to the model's ability to correctly identify unreliable data as unreliable.^50^ Sensitivity and specificity are defined as:

(4) $$\mathrm{Sensitivity}\;=\;\frac{\mathrm{TP}\;}{\mathrm{TP}\;+\;\mathrm{FN}}$$

(5) $$\mathrm{Specificity}\;\;=1-\;\frac{\mathrm{FP}\;}{\mathrm{TN}\;+\mathrm{FP}}$$

where TP are true positives; FN are false negatives; TN are true negatives; and FP are false negatives. The receiver operating characteristic (ROC) curve and area under the curve (AUC) value of the final trained model were reported. The thresholds across all training folds were averaged and selected as a final cutoff threshold. After model training, the LR model was used to test the three held‐out patients; each representing a different level of tracking quality. In addition, the confusion matrices at the selected threshold were calculated for the three held‐out patients.

### 2.5 Generative AI and large language models (LLMs)

OpenAI GPT‐5.1 (ChatGPT interface), 2025 version, was used to assist with drafting portions of the research code and to support manuscript preparation, including editing and organization. Prior to use, all AI‐assisted content was carefully reviewed, validated, and verified by the authors. No identifiable patient information or confidential data were entered into the tool.

## 3 RESULTS

The final parameters and 95% confidence intervals (CIs) of the trained SE‐LR and DE‐LR models are summarized in Table 2. The models were developed using features derived from the template matching algorithm (match score, PSR, *x*‐ and *y*‐velocities), along with a DI to account for differences between phantom (DI = 0) and patient (DI = 1) data. For both the SE‐LR and DE‐LR models, the coefficient (*β*_2_) for the PSR term had the largest positive value. This indicates that a higher PSR strongly correlates with a higher probability of reliable tracking, confirming its role as a key predictor of successful template matches. The *x*‐ and *y*‐velocities generally showed negative coefficients, suggesting that tumors with a higher velocity between frames are associated with a lower probability of reliable tracking. The DI and interaction features capture the performance differences between phantom and patient data. The negative *β*_7_ values for the DI × PSR term in both models (SE: −1.192, DE: −0.695) indicate that the predictive strength of PSR is diminished when transitioning from controlled phantom conditions to more complex patient anatomy. Lastly, the cutoff thresholds were optimized to maintain a specificity of 95% on the training data across all patient folds, a critical requirement for minimizing false positives in a clinical setting. For phantom data, the selected thresholds were 0.536 for SE and 0.591 for DE, while for patient data, the corresponding values were 0.848 and 0.772, respectively.

**TABLE 2:** Final intercepts (*β*_0_), coefficient values of the features (*β*_1_–*β*_9_), thresholds, and AUC along with 95% confidence interval of the trained SE‐LR and DE‐LR models.

Variables	SE	DE
*β*_0_	−0.357 (−0.368, −0.339)	1.177 (1.163, 1.184)
*β*_1_	0.705 (0.691, 0.712)	0.866 (0.852, 0.878)
*β*_2_	2.649 (2.624, 2.651)	3.165 (3.111, 3.157)
*β*_3_	−0.234 (−0.238, −0.231)	−0.236 (−0.239, −0.234)
*β*_4_	0.024 (0.020, 0.027)	−0.347 (−0.350, −0.344)
*β*_5_	2.868 (2.833, 2.871)	1.689 (1.660, 1.700)
*β*_6_	−0.090 (−0.109, −0.079)	0.048 (0.024, 0.058)
*β*_7_	−1.192 (−1.194, −1.156)	−0.695 (−0.691, −0.646)
*β*_8_	−0.266 (−0.268, −0.258)	−0.254 (−0.257, −0.248)
*β*_9_	−0.330 (−0.334, −0.325)	−0.155 (−0.161, −0.150)
Phantom threshold	0.536 (0.529, 0.542)	0.591 (0.587, 0.594)
Phantom AUC	0.894 (0.833, 0.917)	0.971 (0.948, 0.984)
Patient threshold	0.848 (0.846, 0.851)	0.772 (0.770, 0.774)
Patient AUC	0.902 (0.862, 0.926)	0.917 (0.873, 0.931)

AUC, area under the curve; DE, dual‐energy; DI, domain indicator; LR, logistic regression; PSR, peak‐to‐side peak ratio; SE, single‐energy.

**FIGURE 3:**
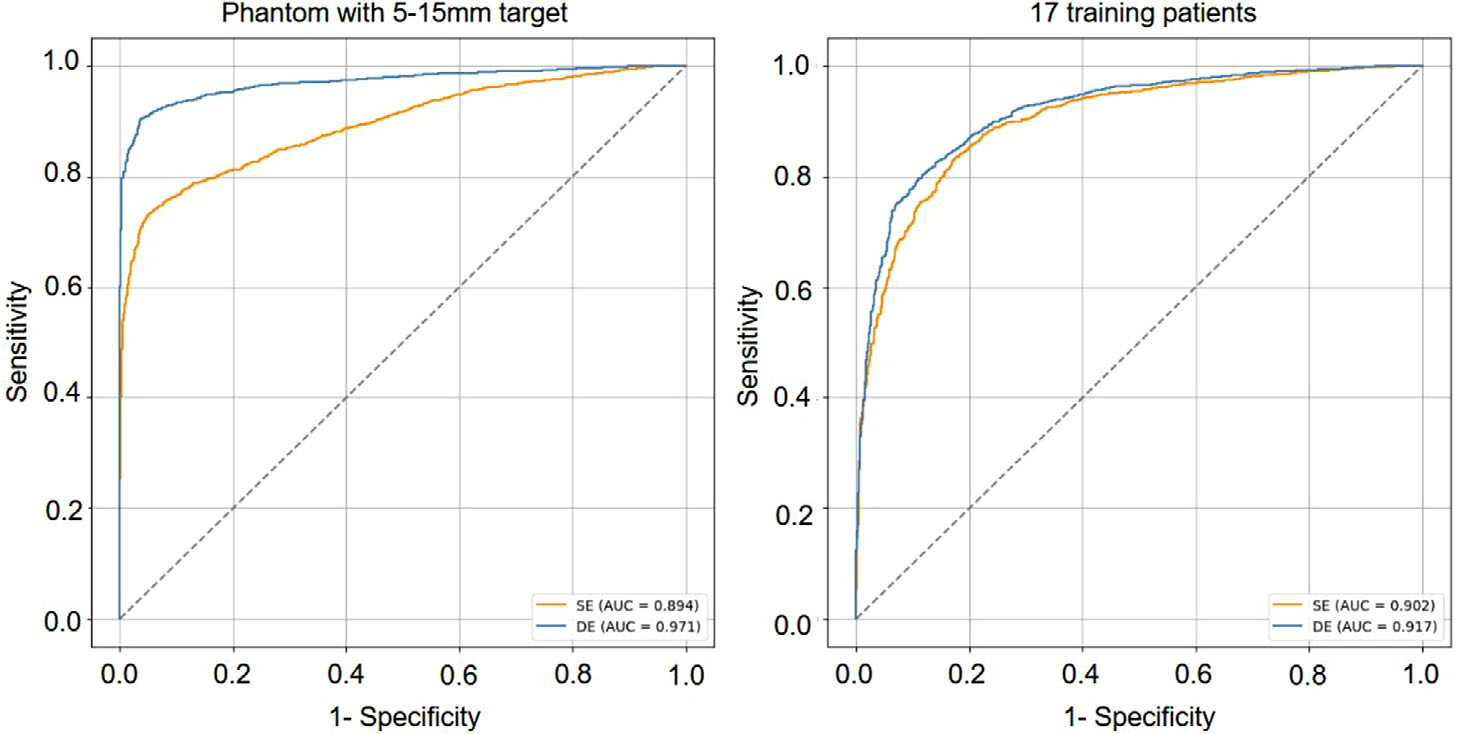
The receiver operating characteristic (ROC) curve results for the single‐energy (SE) and dual‐energy (DE) logistic regression models of phantom (left) and patient (right) data.

Figure 3 presents the ROC curves for the SE‐LR and DE‐LR models as assessed on both phantom and patient data. The AUC serves as a measure of a model's ability to distinguish between reliable and unreliable tracking across all possible classification thresholds. The DE‐LR model consistently outperformed the SE‐LR model across all datasets. Tables 3 and 4 summarize the training data statistics for both phantom and patients. The number of missing frames (MFs) where the algorithm was unable to track the tumor are presented, along with the resulting sensitivity and specificity obtained using the selected cutoff threshold. As discussed previously, the cutoff threshold was chosen to target a specificity of 95%. This high specificity was motivated by clinical considerations, aiming to minimize the risk of misclassifying unreliable tracking as reliable. For phantom data, the average sensitivity of DE‐LR model was significantly higher than that of SE‐LR model (90.8% vs. 73.1%, *p* < 0.01) while maintaining a specificity of 95%. Similarly, the average sensitivity was 65.7% for DE‐LR versus 58.9% for SE‐LR model (*p* < 0.01) for the patient data.

**TABLE 3:** Summary statistics of single energy (SE) and dual energy (DE) images for the phantom training data.

	SE			DE		
Tumor size (mm)	MF	Pred/GT reliable (Sen.)	Pred/GT unreliable (Spe.)	MF	Pred/GT reliable (Sen.)	Pred/GT unreliable (Spe.)
5	61	266/617 (43.1%)	616/622 (99.0%)	42	709/872 (81.3%)	374/392 (95.4%)
10	52	713/944 (75.5%)	281/311 (90.3%)	20	1134/1215 (93.3%)	84/89 (94.3%)
15	48	979/1119 (87.5%)	143/161 (88.8%)	16	1183/1246 (94.9%)	64/69 (92.7%)
Total	161	1958/2680 (73.1%)	1040/1094 (95.0%)	78	3026/3333 (90.8%)	522/550 (94.9%)

GT, ground truth; MF, missing frames; Pred, predicted; Sen., sensitivity; Spe., specificity.

**TABLE 4:** Summary statistics of single energy (SE) and dual energy (DE) images for the patient training data.

		SE			DE		
Patient quality	# Pts.	MF	Pred/eGT reliable (Sen.)	Pred/eGT unreliable (Spe.)	MF	Pred/eGT reliable (Sen.)	Pred/eGT unreliable (Spe.)
Poor	3	14	100/316 (31.6%)	214/224 (95.5%)	196	99/260 (38.1%)	315/328 (96%)
Moderate	7	137	578/1130 (51.2%)	249/264 (94.3%)	119	658/1135 (58%)	259/277 (93.5%)
Good	8	65	1151/1656 (69.5%)	99/103 (96.1%)	60	1236/1636 (75.6%)	121/128 (94.5%)
Total	18	346	1829/3102 (58.9%)	562/591 (95.0%)	275	1993/3031 (65.7%)	695/733 (94.8%)

eGT, estimated ground truth; MF, missing frames; Pred, predicted; Pts, patients; Sen., sensitivity; Spe., specificity.

**FIGURE 4:**
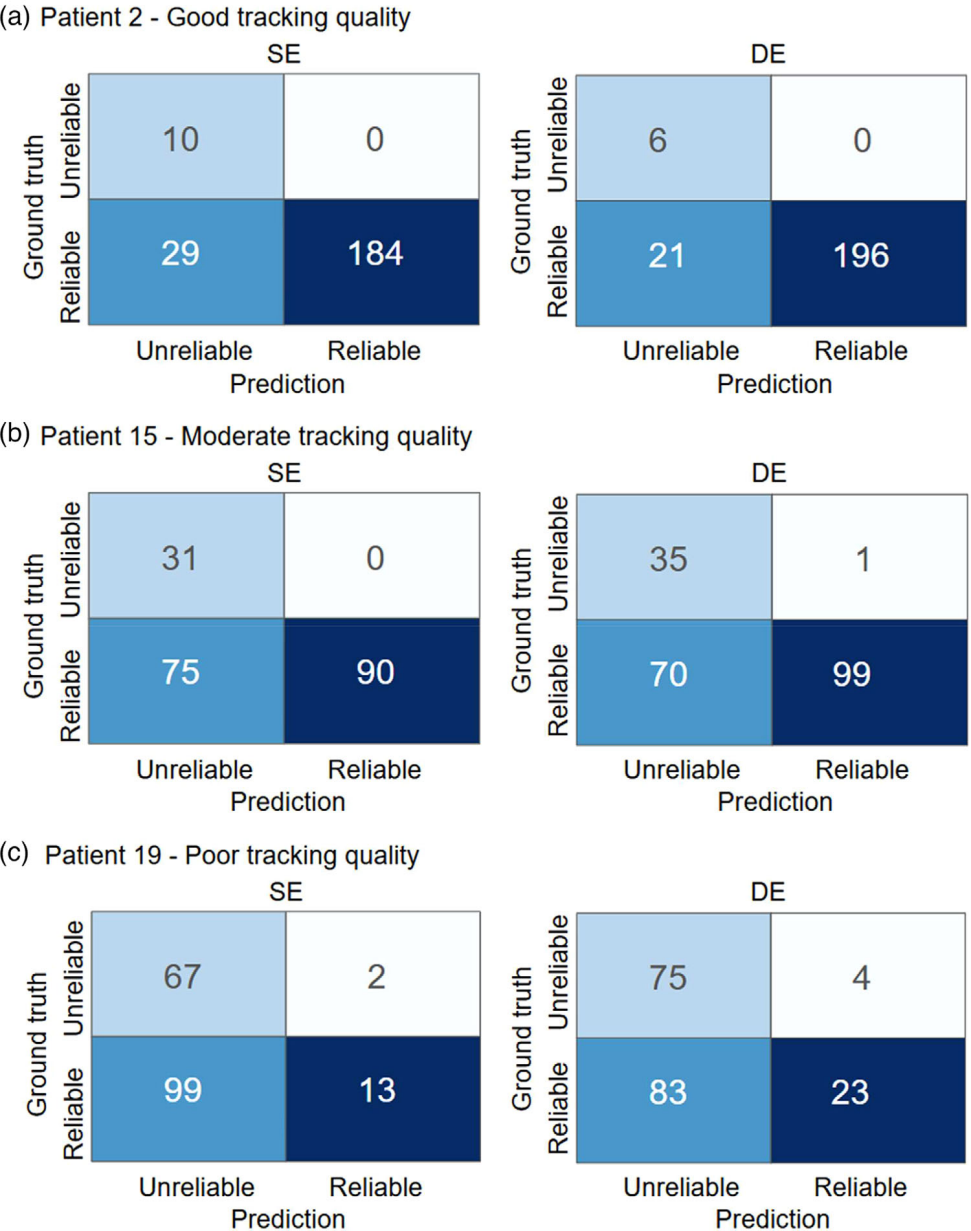
Confusion matrix of (a) Patient 2 – good tracking patient, (b) Patient 15 – moderate tracking patient, and (C) Patient 19 – poor tracking patient.

The final SE‐LR and DE‐LR models were deployed on the three held‐out patients, categorized by tumor tracking quality as good (Pt 2), moderate (Pt 15), and poor (Pt 19). Table 5 provides a summary of the sensitivity and specificity, while Figure 4 presents the confusion matrix of each tested patient. In general, the performance of DE‐LR model was higher than that of the SE‐LR model. Specifically, the patient with good tracking (Pt 2) demonstrated the highest performance metrics for both models, while the patient with poor tracking (Pt 19) exhibited the lowest performance. This suggests that for the high‐quality patient, the trained models can effectively distinguish between reliable and unreliable tracking data. For the patient with moderate tracking (Pt 15), the sensitivity decreased (SE: 0.545, DE: 0.586) even though the specificity remained at ≥95% (SE: 1.000, DE: 0.972).

**TABLE 5:** Sensitivity and specificity of the three held‐out patients.

		SE		DE	
Patient quality	Patient ID	Sen.	Spe.	Sen.	Spe.
Poor	19	0.116	0.971	0.217	0.949
Moderate	15	0.545	1.000	0.586	0.972
Good	2	0.864	1.000	0.903	1.000

Sen., sensitivity; Spe., specificity.

## 4 DISCUSSION

In this study, we produced an ML model to predict the accuracy of MTT using template tracking for both SE and DE imaging. The model was designed using both phantom and patient data. From the tracking results, match score, PSR, *x*‐ and *y*‐velocities were extracted and selected as input features. DI and interaction terms were also incorporated into the model to address the distributional differences between two types of datasets.^37^, ^39^ Sample weighting was applied during training to account for differences in patient tracking quality, ensuring that higher‐quality data had greater influence on the model.^42^ Lastly, hyperparameter tuning was then performed to optimize overall model performance.

Overall, our trained LR models demonstrated that tracking with DE images has a higher sensitivity for a 95% specificity in both phantom and patients. This mirrors previous findings that DE imaging improves tracking success and accuracy due to bone suppression capability.^3^ For SE‐ and DE‐LR models, the performance was notably better on phantom data compared to patient data, which is expected given the controlled nature of the phantom experiments (e.g., ideal tumor shape, fixed material densities, and precise GT) compared to the inherent variability and complexity of patient anatomy and motion.

Using the thresholds determined from the training data, we applied the model to three held‐out patients. For all three cases, the DE‐LR model outperformed the SE‐LR model, although the magnitude of the sensitivities varied across the test patients. This variation in sensitivity is likely attributable to inherent differences in tumor characteristics and location. For example, the patient with good tracking data (Pt 2) had a relatively large tumor located in the upper right lung (RUL), where respiratory motion is relatively limited. This resulted in more consistently successful tracking points and fewer episodes of failed tracking. In contrast, the tumors of Pts 15 and 19, whom had moderate and poor tracking respectively, were in the lower lung, a region subject to greater respiratory motion, making it more challenging for the model to predict reliability. Under these conditions, the model is forced to balance increased sensitivity at the expense of the lower specificity. As a result, sensitivity to true positives was consequently reduced since the selected threshold for classification was set to maintain a fixed specificity of 95%. This indicates that only a limited number of frames were classified as reliable for MTT. In such cases, alternative motion management approaches such as respiratory gating and breath‐hold may be required.

A major challenge in validating MTT performance in the clinical setting is a lack of GT. To address this issue, a number of strategies has been developed. Several studies have eGT using Bayesian frameworks that integrate model predictions with image‐based measurements.^8^, ^24^, ^51^ Shieh et al. proposed a Bayesian workflow to enable the estimation of 3D tumor position using 2D kV imaging for MTT.^8^ For each image frame, the algorithm utilized prior motion knowledge with the likelihood from the 2D image to predict the tumor's 3D location. Although the approach is noninvasive and designed based on temporal correlations, its accuracy is sensitive to image‐based tracking errors and prior inaccuracies, which may bias the estimated trajectories.^8^ Building on these concepts, Nguyen et al. implemented a KF framework for real‐time tumor tracking during image‐guided radiation therapy (IGRT) for prostate cance.^23^ Although KF enables continuous state estimation, in the context of lung cancer radiotherapy, relying solely on KF may be insufficient. KF assumes linear dynamics and Gaussian noise,^25^, ^26^ yet lung tumor motion is often nonlinear, patient‐specific, and influenced by irregular breathing patterns. These complexities can lead to biased or inaccurate KF predictions, highlighting the need for complementary approaches that can assess the reliability of tracking in real time. Another alternative relies on external respiratory surrogates, such as the real‐time position management (RPM, Varian Medical Systems) system, which monitors chest or abdominal surface motion to approximate internal tumor displacement.^7^, ^9^ However, it is worth noting that the reliance on an external surrogate like RPM signals introduces uncertainty due to potential phase shift and amplitude mismatch between external respiratory and internal tumor motion.^52^, ^53^

Given these limitations, there is a growing need for methods that can assess and ensure tracking reliability without relying on implanted markers or external signals. By leveraging the rapid development of AI, we introduced the first ML model designed to evaluate the reliability of template‐based MTT for lung tumor motion management. The proposed LR models produced promising results, addressing essential aspects of MTT validation and clinical implementation.

In general, LR offers a transparent framework that captures feature interactions and provides interpretable insight into the relationships among features.^27^, ^28^ The model produces coefficients that are directly explainable, thus allowing clinicians to see how each input feature contributes to the reliability of MTT. In our case, PSR has the highest coefficient among the input features; thus, it is the most important factor when determining the reliability of the tracking data. This finding aligns with previous studies that also suggest the higher the PSR value, the more reliable the MTT is.^4^, ^29^ Compared to other advanced ML models, LR is more robust to deploy in clinical workflows because it has simpler structure and minimal hyperparameters, leading to lower risk of overfitting, fewer tuning requirements, and higher reproducibility across settings.^54^, ^55^, ^56^

Given that our study aimed to develop a clinically practical and interpretable model, we employed LR as the primary approach, which produced AUC of 0.902 (95% CI: 0.862, 0.926) for SE and 0.917 (95% CI: 0.873, 0.931) for DE in clinical data. Although the study only employed LR models, the preliminary results demonstrated the feasibility of the ML models in evaluating template‐based MTT. It is important to note that the rationale for using LR is supported by prior work,^57^ which showed that more complex ML architectures do not necessarily yield improved predictive performance. In that particular study, the authors developed and compared 13 ML classifiers, such as LR, RF, support vector classifier (SVC), multilayer perceptrons (MLPs), etc., to predict the accuracy of lung and liver tumor motion tracking using radiomic features.^57^ The results indicate that for lung tumors, LR (AUC = 0.941 ± 0.011) and SVC (AUC = 0.941 ± 0.010) achieved the highest predictive performance; for liver tumors, LR (AUC = 0.892 ± 0.020) also demonstrated performance comparable to the MLP (AUC = 0.893 ± 0.022) and SVC (AUC = 0.895 ± 0.021).^57^ Of note, Li et al. used an external surrogate‐based approach to track tumor motion with the RPM system and showed that radiomic features such as tumor intensity, shape, and texture can predict tracking accuracy. In contrast, our study utilized MTT on kV images and demonstrated that the tracking‐intrinsic features derived directly from template‐matching could also serve as effective predictors of tracking reliability. Despite these different methodologies, both studies highlight the potential use of ML as a robust framework for evaluating tumor tracking quality.

The present study has some limitations. First, the sample size of 20 patients is relatively small, which will limit the generalizability and statistical power of the findings. Future research should focus on conducting a prospective clinical study with a larger and more heterogenous cohort to assess the model's performance across diverse patient populations. Given the limited dataset, sample weighting based on TSR was incorporated to reflect confidence in the eGT labels. This strategy was intended to mitigate the potential influence of mislabeled samples, which may have a disproportionate impact in small datasets. However, TSR does not directly quantify the reliability of the GT annotations. Consequently, under‐weighting patients with lower TSR may reduce the contribution of potentially informative samples that contain a broader range of tracking conditions. Future studies with larger datasets should further investigate the impact of alternative weighting strategies, including uniform weighting and dynamic weightin^43^, ^58^ derived from tracking metrics. It is also important to note that the current analysis was performed offline; therefore, the feasibility of implementing the proposed model for online reliability assessment during radiation treatment delivery has not yet been evaluated. Future work should investigate its computational efficiency and performance for deployment in real‐time clinical workflows. Another limitation is that in this study, the feature engineering process is limited to low‐order variables extracted directly from template‐based tracking data, including match score, PSR, and *x*‐ and *y*‐velocities. Incorporating high‐order or transformed features could potentially enhance model performance and provide deeper insights into tracking success.^57^, ^59^

Despite these limitations, the proposed LR models offer a promising approach for evaluating the reliability of template‐based MTT for lung cancer treatment. As demonstrated, the proposed model is highly interpretable and straightforward to implement in clinical settings while still offering promising predictive performance. Another strength is addressing the common challenge that ML models trained solely on phantom data often fail to generalize well to patient data.^60^ By incorporating a DI variable and corresponding interaction terms, the proposed LR models effectively distinguished between phantom and patient domains, allowing it to adapt its learned relationships accordingly. This design enhances model generalizability across datasets and provides an efficient, clinically favorable framework for implementation.

The findings of this study provide a foundation for future research and clinical use. The proposed models have the potential to guide imaging modality selection for MTT during treatment. Although SE imaging is generally the standard for MTT, its performance may degrade in situations where bony structures obscure the tumor. In such cases, DE imaging can offer improved visualization by suppressing bone and enhancing tumor contrast.^18^ The proposed models could provide a quantitative metric for each modality on a frame‐by‐frame basis, allowing MTT algorithm to identify which modality is more trustworthy at each moment. This capability could be integrated into real‐time treatment workflows, supporting decision‐making, and enabling clinicians to select the most reliable modality during therapy.

## 5 CONCLUSION

The study demonstrates that the proposed LR models can be used to evaluate the reliability of MTT on both SE and DE imaging. However, additional clinical data are needed to generalize this type of ML model for the use of MTT prior to the clinical implementation.

## AUTHOR CONTRIBUTIONS

All listed authors contributed to the work and to writing the article.

## ETHICS STATEMENT

The study was approved by the Institutional Review Board (IRB) of Loyola University Chicago (LU208072). Patient data were prospectively collected and analyzed in a de‐identified manner, and the requirement for informed consent was obtained. Phantom experiments did not involve human subjects.

## CONFLICT OF INTEREST STATEMENT

Mathias Lehmann and Liangjia Zhu are employed by Varian Medical Systems.

## Data Availability

Authors are not able to share data at this time.

## References

[1] Haytmyradov M , Mostafavi H , Wang A , et al. Markerless tumor tracking using fast‐kV switching dual‐energy fluoroscopy on a benchtop system. Med Phys. 2019;46(7):3235‐3244. doi:10.1002/mp.13573 31059124 PMC6625841

[2] Roeske JC , Mostafavi H , Haytmyradov M , et al. Characterization of markerless tumor tracking using the on‐board imager of a commercial linear accelerator equipped with fast‐kV switching dual‐energy imaging. Adv Radiat Oncol. 2020;5(5):1006‐1013. doi:10.1016/j.adro.2020.01.008 33089019 PMC7560565

[3] Haytmyradov M , Patel R , Mostafavi H , et al. A novel phantom for characterization of dual energy imaging using an on‐board imaging system. Phys Med Biol. 2019;64(3):1‐20. doi:10.1088/1361-6560/aaf9dd PMC6602048 30566913

[4] Block AM , Patel R , Harkenrider MM , Surucu M , Roeske JC . Dual energy fluoroscopy for markerless motion tracking of lung tumors in stereotactic body radiation therapy (SBRT). Int J Radiat Oncol Biol Phys. 2015;93(3):S41‐S42. doi:10.1016/j.ijrobp.2015.07.101

[5] Roeske JC , Mostafavi H , Lehmann M , et al. Initial clinical evaluation of fast‐kV dual energy imaging for markerless tumor tracking of lung tumors in stereotactic body radiation therapy (SBRT). Int J Radiat Oncol Biol Phys. 2021;111:S47‐S48. doi:10.1016/j.ijrobp.2021.07.128

[6] Mueller M , Poulsen P , Hansen R , et al. The markerless lung target tracking AAPM Grand Challenge (MATCH) results. Med Phys. 2022;49(2):1161‐1180. doi:10.1002/mp.15418 34913495 PMC8828678

[7] de Bruin K , Dahele M , Mostafavi H , Slotman BJ , Verbakel WFAR . Markerless real‐time 3‐dimensional kV tracking of lung tumors during free breathing stereotactic radiation therapy. Adv Radiat Oncol. 2021;6(4):100705. doi:10.1016/j.adro.2021.100705 34113742 PMC8170355

[8] Shieh CC , Caillet V , Dunbar M , et al. A Bayesian approach for three‐dimensional markerless tumor tracking using kV imaging during lung radiotherapy. Phys Med Biol. 2017;62(8):3065‐3080. doi:10.1088/1361-6560/aa6393 28323642 PMC5729104

[9] Grama D , Dahele M , Slotman B , Verbakel WFAR . Explainable AI for raising confidence in deep learning‐based tumor tracking models. Med Phys. 2025;52(7):1‐11. doi:10.1002/mp.17940 PMC12260770 40660895

[10] Grama D , Dahele M , van Rooij W , Slotman BJ , Gupta DK , Verbakel W . Siamese‐based deep learning for markerless lung tumor tracking during stereotactic radiotherapy. Int J Radiat Oncol Biol Phys. 2021;111(3):e111. doi:10.1016/j.ijrobp.2021.07.517

[11] Kelly CJ , Karthikesalingam A , Suleyman M , Corrado G , King D . Key challenges for delivering clinical impact with artificial intelligence. BMC Med. 2019;17(1):1‐9. doi:10.1186/s12916-019-1426-2 31665002 PMC6821018

[12] Zou K , Chen Z , Yuan X , Shen X , Wang M , Fu H . A review of uncertainty estimation and its application in medical imaging. Meta‐Radiology. 2023;1(1):1‐12. doi:10.1016/j.metrad.2023.100003

[13] Yuan X , Ma C , Hu M , et al. Machine learning in image‐based outcome prediction after radiotherapy: a review. J Appl Clin Med Phys. 2025;26(1):1‐25. doi:10.1002/acm2.14559 PMC11712300 39556691

[14] Field M , Hardcastle N , Jameson M , Aherne N , Holloway L . Machine learning applications in radiation oncology. Phys Imaging Radiat Oncol. 2021;19(June):13‐24. doi:10.1016/j.phro.2021.05.007 34307915 PMC8295850

[15] Fu Y , Zhang P , Fan Q , et al. Deep learning‐based target decomposition for markerless lung tumor tracking in radiotherapy. Med Phys. 2024;51(6):4271‐4282. doi:10.1002/mp.17039 38507259 PMC12123686

[16] Grama D , Dahele M , van Rooij W , Slotman B , Gupta DK , Verbakel WFAR . Deep learning‐based markerless lung tumor tracking in stereotactic radiotherapy using Siamese networks. Med Phys. 2023;50(11):6881‐6893. doi:10.1002/mp.16470 37219823

[17] Yan Y , Fujii F , Shiinoki T . Marker‐less lung tumor tracking from real‐time color x‐ray fluoroscopic images using cross‐patient deep learning model. Bioengineering. 2025;12(11):1‐29. doi:10.3390/bioengineering12111197 PMC12649688 41301153

[18] Haytmyradov M , Mostafavi H , Morf D , et al. Markerless tumor tracking using fast‐kV switching dual energy imaging with the on‐board imager of a commercial linac. Int J Radiat Oncol Biol Phys. 2019;105(1):S29‐S30. doi:10.1016/j.ijrobp.2019.06.438 PMC7560565 33089019

[19] Luce J , Kaur M , Dingillo J , et al. Use of a deep learning neural network to generate bone suppressed images for markerless lung tumor tracking. Med Phys. 2025;52(7):1‐13. doi:10.1002/mp.17949 PMC12260777 40660921

[20] Kaur M , Wagstaff P , Mostafavi H , et al. Effect of different noise reduction techniques and template matching parameters on markerless tumor tracking using dual‐energy imaging. J Appl Clin Med Phys. 2022;23(12):e13821. doi:10.1002/acm2.13821 PMC9797162 36350280

[21] Kaur M , Luce J , Lehmann M , et al. Effect of scattered megavoltage x‐rays on markerless tumor tracking using dual energy kilovoltage imaging. J Appl Clin Med Phys. 2023;24(8):e13993. doi:10.1002/acm2.13993 PMC10402669 37071500

[22] Mostafavi H , Sloutsky A , Jeung A . Detection and localization of radiotherapy targets by template matching. In Proceedings of the Annual International Conference of the IEEE Engineering in Medicine and Biology Society . EMBS; 2012:6023‐6027. doi:10.1109/EMBC.2012.6347367 23367302

[23] Nguyen DT , Keall P , Booth J , Shieh CC , Poulsen P , O'Brien R . A real‐time IGRT method using a Kalman filter framework to extract 3D positions from 2D projections. Phys Med Biol. 2021;66(21). doi:10.1088/1361-6560/ac06e3 34062512

[24] Kalman RE . A new approach to linear filtering and prediction problems. J Fluids Eng Trans ASME. 1960;82(1):35‐45. doi:10.1115/1.3662552

[25] Jiang S , Shi J , Moura S . A New Framework for Nonlinear Kalman Filters. 2024:1‐15. http://arxiv.org/abs/2407.05717

[26] Budhiraja A , Chen L , Lee C . A survey of numerical methods for nonlinear filtering problems. Phys D. 2007;230(1‐2):27‐36. doi:10.1016/j.physd.2006.08.015

[27] Lu SC , Swisher CL , Chung C , Jaffray D , Sidey‐Gibbons C . On the importance of interpretable machine learning predictions to inform clinical decision making in oncology. Front Oncol. 2023;13(February):1‐13. doi:10.3389/fonc.2023.1129380 PMC10013157 36925929

[28] Uddin S , Khan A , Hossain ME , Moni MA . Comparing different supervised machine learning algorithms for disease prediction. BMC Med Inform Decis Mak. 2019;19(1):1‐16. doi:10.1186/s12911-019-1004-8 31864346 PMC6925840

[29] Roeske JC , Mostafavi H , Block AM , et al. Dual energy fluoroscopy improves the accuracy of template‐based tracking in lung cancer patients receiving stereotactic body radiation therapy. Int J Radiat Oncol Biol Phys. 2017;99(2):S94‐S95. doi:10.1016/j.ijrobp.2017.06.228

[30] Thong YK , Woolfson MS , Crowe JA , Hayes‐Gill BR , Jones DA . Numerical double integration of acceleration measurements in noise. Measurement (Lond). 2004;36(1):73‐92. doi:10.1016/j.measurement.2004.04.005

[31] van Breugel F , Kutz JN , Brunton BW . Numerical differentiation of noisy data: a unifying multi‐objective optimization framework. IEEE Access. 2020;8:196865‐196877. doi:10.1109/ACCESS.2020.3034077 33623728 PMC7899139

[32] Pinheiro JMH , de Oliveira SVB , Silva THS , et al. The Impact of Feature Scaling In Machine Learning: Effects on Regression and Classification Tasks. 2025. http://arxiv.org/abs/2506.08274

[33] Borm KJ , Oechsner M , Wiegandt M , Hofmeister A , Combs SE , Duma MN . Moving targets in 4D‐CTs versus MIP and AIP: comparison of patients data to phantom data. BMC Cancer. 2018;18(1):1‐9. doi:10.1186/s12885-018-4647-4 30041618 PMC6056919

[34] Faddegon B , Descovich M , Chen K , et al. A digital male pelvis phantom series showing anatomical variations over the course of fractionated radiotherapy treatment. Med Phys. 2024;51(4):3034‐3044. doi:10.1002/mp.16865 38071746

[35] Li JL , Hsu CF , Chang MC , Chen WC . A Comprehensive Review of Machine Learning Advances on Data Change: A Cross‐Field Perspective. 2024;XX(X):1‐20. http://arxiv.org/abs/2402.12627

[36] Guan H , Liu M . Domain adaptation for medical image analysis: a survey. IEEE Trans Biomed Eng. 2022;69(3):1173‐1185. doi:10.1109/TBME.2021.3117407 34606445 PMC9011180

[37] Gao Y , Bao R , Ji Y , et al. Transfer Learning with Clinical Concept Embeddings from Large Language Models. 2024:167‐176. http://arxiv.org/abs/2409.13893 PMC12150738 40502269

[38] Guan H , Liu M . DomainATM: domain adaptation toolbox for medical data analysis. Neuroimage. 2023;268:1‐10. doi:10.1016/j.neuroimage.2023.119863 10.1016/j.neuroimage.2023.119863 PMC9908850 36610676

[39] Jaccard J . Interaction Effects in Logistic Regression (Quantitative Applications in the Social Sciences). SAGE Publications, Inc; 2001.

[40] Wang JC , Huan SK , Kuo JR , Lu CL , Lin H , Shen KH . A multivariable logistic regression equation to evaluate prostate cancer. J Formos Med Assoc. 2011;110(11):695‐700. doi:10.1016/j.jfma.2011.09.005 22118313 10.1016/j.jfma.2011.09.005

[41] Hosmer DW , Lemeshow S , Sturdivant RX . Applied Logistic Regression. John Wiley & Sons, Inc.; 2013.

[42] Yi G , Liu X , Ma L , Han M , Niu L . Dynamic learning of sample ambiguity‐driven sample weighting for medical image classification. Expert Syst Appl. 2026;298:129527. doi:10.1016/j.eswa.2025.129527

[43] Liu K , Liu J , Liu S . Enhanced semi‐supervised medical image classification based on dynamic sample reweighting and pseudo‐label guided contrastive learning (DSRPGC). Mathematics. 2024;12(22):1‐29. doi:10.3390/math12223572

[44] Hauptmann T , Fellenz S , Nathan L , Tüscher O , Kramer S . Discriminative machine learning for maximal representative subsampling. Sci Rep. 2023;13(1):1‐13. doi:10.1038/s41598‐023‐48177‐3 38017053 10.1038/s41598-023-48177-3 PMC10684887

[45] Arafa A , Radad M , Badawy M , El‐Fishway N . Logistic regression hyperparameter optimization for cancer classification. Menoufia J Electron Eng Res. 2022;31(1):1‐8. doi:10.21608/mjeer.2021.70512.1034

[46] Książek W , Gandor M , Pławiak P . Comparison of various approaches to combine logistic regression with genetic algorithms in survival prediction of hepatocellular carcinoma. Comput Biol Med. 2021;134:104431. doi:10.1016/j.compbiomed.2021.104431 10.1016/j.compbiomed.2021.104431 34015670

[47] Zou H , Hastie T . Regularization and variable selection via the elastic net. J R Stat Soc Series B Stat Methodol. 2005;67(2):301‐320. doi:10.1111/j.1467‐9868.2005.00503.x

[48] Tougui I , Jilbab A , Mhamdi JE . Impact of the choice of cross‐validation techniques on the results of machine learning‐based diagnostic applications. Healthc Inform Res. 2021;27(3):189‐199. doi:10.4258/HIR.2021.27.3.189 34384201 10.4258/hir.2021.27.3.189 PMC8369053

[49] Bradshaw TJ , Huemann Z , Hu J , Rahmim A . A guide to cross‐validation for artificial intelligence in medical imaging. Radiol Artif Intell. 2023;5(4):e220232. doi:10.1148/ryai.220232 10.1148/ryai.220232 PMC10388213 37529208

[50] Swift A , Heale R , Twycross A . What are sensitivity and specificity? Evid Based Nurs. 2020;23(1):2‐5. doi:10.1136/ebnurs‐2019‐103225 31719126 10.1136/ebnurs-2019-103225

[51] Li R , Fahimian BP , Xing LA . Bayesian approach to real‐time 3D tumor localization via monoscopic x‐ray imaging during treatment delivery. Med Phys. 2011;38(7):4205‐4214. doi:10.1118/1.3598435 21859022 10.1118/1.3598435 PMC3145219

[52] Wang G , Song X , Li G , et al. Correlation of optical surface respiratory motion signal and internal lung and liver tumor motion: a retrospective single‐center observational study. Technol Cancer Res Treat. 2022;21:1‐11. doi:10.1177/15330338221112280 10.1177/15330338221112280 PMC9272160 35791642

[53] Hoffmans D , Remmerts de Vries I , Dahele M , Verbakel W . Quantifying the dosimetric accuracy of expiration‐gated stereotactic lung radiotherapy. Med Phys. 2025;52(5):2773‐2784. doi:10.1002/mp.17743 40091507 10.1002/mp.17743 PMC12059520

[54] Christodoulou E , Ma J , Collins GS , Steyerberg EW , Verbakel JY , Van Calster B . A systematic review shows no performance benefit of machine learning over logistic regression for clinical prediction models. J Clin Epidemiol. 2019;110:12‐22. doi:10.1016/j.jclinepi.2019.02.004 30763612 10.1016/j.jclinepi.2019.02.004

[55] Elgeldawi E , Sayed A , Galal AR , Zaki AM . Hyperparameter tuning for machine learning algorithms used for Arabic sentiment analysis. Informatics. 2021;8(4):1‐21. doi:10.3390/informatics8040079

[56] Shipe ME , Deppen SA , Farjah F , Grogan EL . Developing prediction models for clinical use using logistic regression: an overview. J Thorac Dis. 2019;11(suppl 4):S574‐S584. doi:10.21037/jtd.2019.01.25 31032076 10.21037/jtd.2019.01.25 PMC6465431

[57] Li G , Zhang X , Song X , et al. Machine learning for predicting accuracy of lung and liver tumor motion tracking using radiomic features. Quant Imaging Med Surg. 2023;13(3):1605‐1618. doi:10.21037/qims‐22‐621 36915317 10.21037/qims-22-621 PMC10006135

[58] Xue C , Dou Q , Shi X , Chen H , Heng PA . Robust learning at noisy labeled medical images: applied to skin lesion classification. In Proceedings—International Symposium on Biomedical Imaging . 2019:1280‐1283. doi:10.1109/ISBI.2019.8759203

[59] Ge G , Zhang JZ , Zhang J . The impact of high‐order features on performance of radiomics studies in CT non‐small cell lung cancer. Clin Imaging. 2024;113(July):110244. doi:10.1016/j.clinimag.2024.110244 39096890 10.1016/j.clinimag.2024.110244

[60] Camagni F , Nakas A , Parrella G , et al. Generation of multimodal realistic computational phantoms as a test‐bed for validating deep learning‐based cross‐modality synthesis techniques. Med Biol Eng Comput. 2025; 64: 263‐284. doi:10.1007/s11517‐025‐03437‐4 41015634 10.1007/s11517-025-03437-4 PMC12868042

